# Reducing the use of oral psychotropic PRN medication in acute mental health inpatients

**DOI:** 10.1192/bjo.2021.561

**Published:** 2021-06-18

**Authors:** Zoe Moore, Rachel Morrow, Meta McGee

**Affiliations:** Belfast Health and Social CareTrust

## Abstract

**Aims:**

Project aim:

To reduce the use of oral psychotropic PRN* medication on Ward 3 AMHIC (Acute Mental Health Inpatient Centre) by 20% by May 2020

(*PRN = Pro re nata/As required)

On Ward 3, we identified a number of unintended negative consequences of PRN medication to both patients and staff.

These included issues with over-use, dependence and side effects; as well as loss of staff ownership and challenging interactions with patients, (including escalation to aggression).

Following the success of our Child and Adolescent Mental Health Inpatient colleagues in this area, we decided to embark on a project to change practice within our ward.

**Method:**

In order to quantify the problem, we first collected baseline data on current use of psychotropic PRN medication.

As a multidisciplinary project team, we then brainstormed potential contributory factors and displayed these visually as a driver diagram.

This divided our project into 3 main areas:

1. Safe prescribing
2. Safe administration,
3. Safety culture.

Project measures were also agreed as follows:

Outcome: Number of doses of oral psychotropic PRN medication administered per week

Balancing: Violent incidents; IM administrations of psychotropic medication

Process: Time taken to complete interventions; Patient and staff satisfaction. Change ideas were selected and implemented sequentially, using Plan-Do-Study- Act methodology.

These included:

1. Weekly review of PRN prescribing
2. Nursing administration sheet

Data were collected weekly and plotted on our run chart.

**Result:**

By the end of May 2020, we had exceeded our initial goal, reducing the weekly median number of doses of oral psychotropic PRN medication administered by over 30%.

Our balancing measures remained stable and we gained useful insights and development ideas from a staff survey.

Further change ideas were planned for implementation over the months that followed, however, the impact of the COVID-19 pandemic meant that the project lost some momentum.

**Conclusion:**

Despite running into some difficulty over recent months, the team remain motivated to maintain and build upon our previous success.

In the past few weeks, “Calm Cards”, (a patient-centred intervention promoting use of individualised alternative coping strategies), have been introduced.

We hope that the outcomes of this intervention will be positive, both in terms of further reducing use of PRN medication and encouraging development of skills which can be utilised beyond the hospital environment.

We also intend to share our learning with colleagues and explore the possibility of introducing the project to other wards within the hospital.

